# Lateral wedge shoe insoles for medial knee osteoarthritis: a 12-month randomised controlled trial

**DOI:** 10.1186/1757-1146-4-S1-O7

**Published:** 2011-05-20

**Authors:** Kim L Bennell, Kelly-Ann Bowles, Craig Payne, Flavia Cicuttini, Elizabeth Williamson, Andrew Forbes, Fahad Hanna, Miranda Davies- Tuck, Rana S Hinman

**Affiliations:** 1 Centre for Health, Exercise and Sports Medicine, Department of Physiotherapy, The University of Melbourne, Victoria, Australia; 2 Department of Podiatry & Musculoskeletal Research Centre, LaTrobe University, Melbourne, Australia; 3 Department of Epidemiology and Preventive Medicine, Alfred Hospital, Monash University, Melbourne, Australia; 4 Health Economics Unit, Monash University, Melbourne, Australia; 5 Baker IDI Heart and Diabetes Institute, Commercial Road, Melbourne, Victoria 3004, Australia

## Background

The majority of clinical guidelines recommend lateral wedge shoe insoles for medial knee osteoarthritis (OA), despite limited and equivocal evidence of efficacy. The objective of this study was to assess efficacy of lateral wedge insoles for improving symptoms and slowing structural disease progression compared with control insoles in medial knee OA.

## Methods

A randomised participant- and assessor-blinded controlled trial was used. 200 people aged 50 or more with clinical and radiographic diagnosis of mild-to-moderately severe medial knee OA were recruited. The interventions consisted of full-length 5° lateral wedged insoles or flat control insoles worn inside the shoes daily for 12 months. The primary symptomatic outcome was change in overall knee pain (past week) measured on an 11-point numeric rating scale and primary structural outcome was change in medial tibial cartilage volume from magnetic resonance imaging. Secondary clinical outcomes included changes in measures of pain, function, stiffness, and health-related quality of life. Secondary structural outcomes included progression of medial cartilage defects and bone marrow lesions.

## Results

There were no significant between-group differences for the primary outcomes of change in overall pain (-0.3 points 95% CI (-1.0 to 0.3)) and change in medial tibial cartilage volume (-0.4 mm^3^ (-15.4 to 14.6)). None of the changes in secondary outcomes demonstrated differences between groups.

## Conclusion

In this study, lateral wedge insoles worn for 12 months provided no symptomatic or structural benefits compared to a flat control insole.

